# A comparison of age-standardised event rates for acute and chronic coronary heart disease in metropolitan and regional/remote Victoria: a retrospective cohort study

**DOI:** 10.1186/s12889-016-3081-2

**Published:** 2016-05-11

**Authors:** Paul D. Xanthos, Brett A. Gordon, Stephen Begg, Voltaire Nadurata, Michael I. C. Kingsley

**Affiliations:** Discipline of Exercise Physiology, La Trobe Rural Health School, La Trobe University, Bendigo, Victoria Australia; La Trobe Rural Health School, La Trobe University, Bendigo, Victoria Australia; Department of Cardiology, Bendigo Health Care Group, Bendigo, Victoria Australia

**Keywords:** Cardiac disease, Health services, Public health, Myocardial infarction, Angina

## Abstract

**Background:**

Acute and chronic coronary heart disease (CHD) pose different burdens on health-care services and require different prevention and treatment strategies. Trends in acute and chronic CHD event rates can guide service implementation. This study evaluated changes in acute and chronic CHD event rates in metropolitan and regional/remote Victoria.

**Methods:**

Victorian hospital admitted episodes with a principal diagnosis of acute CHD or chronic CHD were identified from 2005 to 2012. Acute and chronic CHD age-standardised event rates were calculated in metropolitan and regional/remote Victoria. Poisson log-link linear regression was used to estimate annual change in acute and chronic CHD event rates.

**Results:**

Acute CHD age-standardised event rates decreased annually by 2.9 % (95 % CI, −4.3 to −1.4 %) in metropolitan Victoria and 1.7 % (95 % CI, −3.2 to −0.1 %) in regional/remote Victoria. In comparison, chronic CHD age-standardised event rates increased annually by 4.8 % (95 % CI, +3.0 to +6.5 %) in metropolitan Victoria and 3.1 % (95 % CI, +1.3 to +4.9 %) in regional/remote Victoria. On average, age-standardised event rates for regional/remote Victoria were 30.3 % (95 % CI, 23.5 to 37.2 %) higher for acute CHD and 55.3 % (95 % CI, 47.1 to 63.5 %) higher for chronic CHD compared to metropolitan Victoria from 2005 to 2012.

**Conclusion:**

Annual decreases in acute CHD age-standardised event rates might reflect improvements in primary prevention, while annual increases in chronic CHD age-standardised event rates suggest a need to improve secondary prevention strategies. Consistently higher acute and chronic CHD age-standardised event rates were evident in regional/remote Victoria compared to metropolitan Victoria from 2005 to 2012.

## Background

Coronary heart disease (CHD) is the leading cause of death and a significant contributor to health-care expenditure in Australia and around the world [1, 2]. CHD accounted for more than $2 billion of health-care expenditure in Australia in 2008/09, of which 75 % was directed towards hospital-admitted patient services [3]. The appropriate management protocols for individuals with CHD are well established. However, these are dependent on there being available facilities within health-care institutions [4]. Moreover, patients need to recognise the requirement for treatment, be able to access treatment facilities and comply with treatment plans.

Acute CHD, angina pectoris or acute myocardial infarction (AMI), and chronic CHD pose different burdens on health-care services. Primary prevention programs are designed to reduce acute CHD development and secondary prevention programs are designed to treat and manage the progression of chronic CHD [5]. Given this, it is important to identify the incidence of different types of CHD in order to best inform where CHD health-care expenditure should be focused. However, there is a paucity of evidence investigating the incidence of both acute CHD and chronic CHD because the majority of international studies have investigated the incidence of AMI only or a definition of CHD that encompasses all of angina pectoris, AMI and chronic CHD [6–9].

The geographic presentation of CHD events provides important information for CHD service providers. Residents of regional/remote Australia are at a greater risk of CHD due to higher prevalence of tobacco use, alcohol consumption, physical inactivity, and obesity compared to metropolitan Australia [10]. Similarly, higher tobacco use, alcohol consumption and obesity is present in regional/remote residents of the United States [11, 12] and Canada [13] when compared to metropolitan or urban residents. Furthermore, regional/remote areas of Australia are less well-prepared than metropolitan areas for responding to cardiac events. Time to treatment is of vital importance during an acute cardiac event [14], and individuals living in regional/remote areas of Australia [14] as well as the United States [15] are required to travel greater distances to access cardiac services. The prognosis of regional/remote dwellers suffering an acute cardiac event is, therefore, likely to be poorer relative to individuals living in metropolitan areas. As such, in order to determine the suitability of future CHD service provision, it is important to identify patterns in geographical location of CHD. Furthermore, if the higher prevalence of risk factors in regional/remote Australia truly manifests itself as greater CHD event rates in regional/remote Victoria, it might be that service provision for CHD should evolve to accommodate the greater travel distance to cardiac services and higher CHD rates faced by regional/remote residents.

The aims of this study were to investigate the event rates of acute and chronic CHD requiring hospitalisation and acute treatment in Victoria from 2005 to 2012 and to identify differences in acute and chronic CHD event rates between metropolitan and regional/remote locations.

## Methods

### Setting

Victoria is a state in the south-eastern corner of mainland Australia. While a relatively small state in geographical area (sixth largest geographical area of the eight Australian states and territories) [16], it is the second most populous state in Australia with a population of 5.5 million people recorded in the 2011 census, which represents 24.7 % of Australia’s population [17]. The majority of Victoria’s population (4.2 million) is located in the metropolitan regions incorporating the capital city of Melbourne as well as the smaller cities of Geelong and the Mornington Peninsula [18]. The remaining 23.9 % (1.3 million) of the Victorian population are located in areas classified as either regional or remote Australia [18].

### Data acquisition and transformation

Ethics approval for this study was obtained from La Trobe University Human Ethics Committee (ref: 14/125) and approval to access event admission data from the Victorian Admitted Episodes Dataset (VAED) was granted by the Victorian Department of Health. Admission data from the VAED at the local government area level (LGA) from 2005 to 2012 for each episode of care (event) resulting from an acute or chronic CHD event were sourced [19]. Events were classified according to the International Statistical Classification of Diseases and Related Health Problems, Tenth Revision, Australian Modification (ICD-10-AM) codes, I20, I21 and I25 and stratified by either a principal diagnosis of acute CHD (angina or AMI) or chronic CHD [19].

Events were stratified by LGA into two remoteness areas, ‘metropolitan Victoria’ and ‘regional/remote Victoria’ according to the Australian Statistical Geographic Standard Correspondences, July 2011 [20]. In the situation where a LGA was partly classified as one remoteness area and partly classified as another remoteness area, it was classified into the remoteness area that encompassed the majority of the LGA. The regional/remote Victoria remoteness area was an amalgamation of all the individual remoteness areas not classified as ‘major cities of Australia’ due to the small number of events within those individual remoteness areas.

### Statistical analysis

Age-standardised rates with 95 % confidence intervals (95 % CI) were calculated for acute CHD and chronic CHD events in metropolitan Victoria and regional/remote Victoria annually from 2005 to 2012. Rates were standardised using the direct method to the July 2013 Victorian population [21]. Poisson log-link linear regression was used to investigate the estimated annual change in acute CHD and chronic CHD event rates. Models for calculating annual percent change in acute CHD and chronic CHD event rates including year (continuous variable), 10-year age categories (categorical variable) and sex (categorical variable) were calculated using the exponential of the β-coefficient for year. An alpha value of 0.05 was set for statistical significance. Data analysis was completed using IBM SPSS Statistics for Windows (Version 22; IBM Corp, Armonk, NY).

## Results

A total of 163,895 events with a principal diagnosis of acute or chronic CHD from 2005 to 2012 were identified.

After controlling for age and sex, there were annual decreasing trends for acute CHD age-standardised event rates in metropolitan (*p* < 0.001) and regional/remote (*p* = 0.039) Victoria. An estimated -2.9 % (95 % CI, −4.3 to −1.4 %) annual change in acute CHD age-standardised event rates were found in metropolitan Victoria while a −1.7 % (95 % CI, −3.2 to −0.1 %) annual change in acute CHD age-standardised event rates were found in regional/remote Victoria. In contrast, there was an annual increasing trend in chronic CHD age-standardised event rates in both metropolitan (*p* < 0.001) and regional/remote (*p* = 0.001) Victoria when age and sex were held constant. Chronic CHD age-standardised event rates were estimated to increase by 4.8 % (95 % CI, +3.0 to +6.5 %) annually in metropolitan Victoria and 3.1 % (95 % CI, +1.3 to +4.9 %) annually in regional/remote Victoria.

**Fig. 1:**
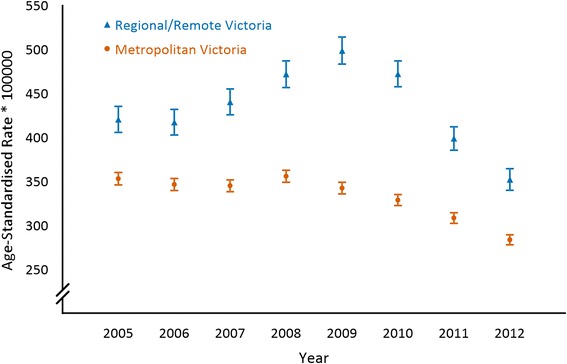
Acute coronary heart disease age-standardised event rates (95 % confidence intervals) in metropolitan and regional/remote Victoria from 2005 to 2012

**Fig. 2:**
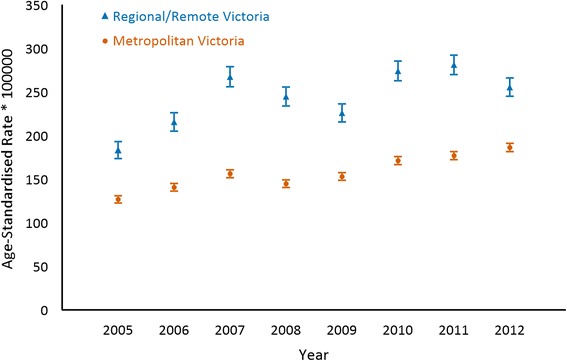
Chronic coronary heart disease age-standardised event rates (95 % confidence intervals) in metropolitan and regional/remote Victoria from 2005 to 2012

Figure 1 shows acute CHD age-standardised event rates (95 % CI) in metropolitan and regional/remote Victoria from 2005 to 2012, which were on average 30.3 % (95 % CI, 23.5 to 37.2 %) higher in regional/remote Victoria compared to metropolitan Victoria. Figure 2 shows chronic CHD age-standardised event rates (95 % CI) in metropolitan and regional/remote Victoria from 2005 to 2012, which was on average 55.3 % (95 % CI, 47.1 to 63.5 %) higher in regional/remote Victoria compared to metropolitan Victoria.

## Discussion

The main findings demonstrate that acute CHD age-standardised event rates have decreased and chronic CHD age-standardised event rates have increased throughout metropolitan and regional/remote Victoria from 2005 to 2012. Both acute and chronic CHD age-standardised event rates were consistently higher in regional/remote Victoria compared to metropolitan Victoria.

While many published studies have investigated the changing incidence of AMI, relatively few studies have investigated the changing trends in acute CHD incidence encompassing both angina and AMI. The results of this study are in agreement with previous studies investigating the age-standardised incidence rates of acute coronary syndromes (synonymous with acute CHD) in Canada [6], the United States [22] and Western Australia [23]. For individuals aged 20 years or older in Canada, the hospitalisation rate for acute coronary syndromes decreased by −3.87 % (95 % CI, −3.86 to −3.88 %) annually from 1994 to 2005. Similarly, a 4 % annual decrease in the acute coronary syndrome discharge rate was found in the United States from 1996 to 2001 [22]. In Western Australia, the age-standardised incidence of acute coronary syndromes for 35 to 84 year olds decreased by −1.7 % (95 % CI, −2.1 to −1.3 %) annually in men and by −1.6 % (95 % CI, −2.1 to −1.0 %) annually in women from 1996 to 2007 [23].

The decline in acute coronary syndromes in these studies has been linked with marked declines in the incidence of unstable angina, with the decline in AMI incidence playing a lesser role in the overall decline in acute coronary syndromes [22, 23]. It has been suggested that the introduction of troponin testing, a sensitive cardiac biomarker, in the late 1990s/early 2000s for the diagnosis of AMI has attenuated the decline in incidence and hospitalisation rates of AMI by simultaneously shifting the diagnoses of unstable angina to AMI [23]. However, given the classification of acute CHD in this study including angina and myocardial infarction, the introduction of troponin testing does not explain the annual decreases in acute CHD shown in this study. Given the decrease in total acute CHD age-standardised event rates and the reduction in acute CHD death rates [2, 24] an alternative explanation is an improvement in the efficacy of primary prevention service provision for acute CHD. This would theoretically result in decreased acute CHD event rates via widespread improvement of the risk factors associated with CHD development.

In contrast to acute CHD, chronic CHD age-standardised event rates increased on an annual basis from 2005 to 2012 in this study. In the Netherlands from 1998 to 2007, researchers found annual increasing trends in chronic CHD hospitalisation rates of 0.7 % (95 % CI, 0.6 to 0.8 %) in men and 2.1 % (95 % CI, 2.0 to 2.2 %) in women [25]. These results, while suggesting a slower rate, reflect the findings of the current study that chronic CHD age-standardised event rates significantly increased annually in both metropolitan and regional/remote Victoria. The increase in chronic CHD age-standardised event rates might also be explained by relatively poor referral and uptake rates for secondary prevention services (cardiac rehabilitation) [26, 27], given that secondary prevention services are designed to treat and manage the development of chronic CHD [5].

Geographic differences in age-standardised event rates in Victoria aligns with previous findings in Quebec [28]. From 1995 to 1997, in individuals 25 years or older, there was a significantly lower AMI incidence rate of 7.21 per 1000 people in the central metropolitan areas compared to small urban centres (8.34 per 1000 people), strong metropolitan influenced zones (7.68 per 1000 people), moderate metropolitan influenced zones (8.10 per 1000 people) and low metropolitan influenced zones (9.26 per 1000 people) [28]. Interestingly, data from Scotland has shown a significantly lower relative risk of CHD in remote small towns and rural areas compared to urban areas [29]. Data from New South Wales, Australia from 1991/92 to 1995/96 showed a significantly higher relative risk for AMI admission of 1.25 (95 % CI, 1.18 to 1.33) for males and 1.43 (95 % CI, 1.35 to 1.54) for females aged between 35 and 74 residing in rural and remote areas compared to individuals residing in the capital city after adjusting for age [30]. Furthermore, the researchers also found significantly higher relative risks for CHD mortality in males and females residing in rural and remote New South Wales over a similar period [30]. Nationwide data suggested a significantly higher hospitalisation rate due to CHD with increasing remoteness Australia-wide in the 2007/08 financial year [2]. The current study is consistent with these findings, which highlight the influence of remoteness on AMI admission [30] and CHD hospitalisation [2]. In addition, the current study extends previous findings by adding angina events to AMI events, and separating them from chronic CHD age-standardised event rates; when this classification was adopted there were consistently higher acute CHD and chronic CHD age-standardised event rates in regional/remote Victoria compared to metropolitan Victoria from 2005 to 2012.

Differential access to appropriate cardiac health-care services is one factor that might explain the differences in acute and chronic CHD age-standardised event rates between metropolitan and regional/remote Victoria. Individuals residing in metropolitan areas have greater access to cardiac health-care services than individuals living in regional/remote areas [14]. As such, individuals living in regional/remote areas tend to require longer commutes in order to access appropriate health-care services [31]. Therefore, longer travel times for individuals requiring cardiac facilities in regional/remote Victoria might result in individuals being either unwilling or unable to receive necessary treatment for their cardiac disease or risk factors of cardiac disease, thereby increasing the acute and chronic CHD age-standardised event rates in regional/remote Victoria.

There are two main limitations to the present study. Firstly, the VAED only provided data on episodes of care from acute and chronic CHD events and record linkage was not permitted; consequently, an individual with multiple admissions for CHD would be included more than once in these analyses. In Western Australia from 1995 to 2005, 36 % of individuals with a nonfatal AMI from 35 to 84 years of age had previously established CHD, highlighting the potential impact of double-counting in this study [32]. However, this limitation was unavoidable given that record linkage was not permitted. Secondly, although admission codes relating to CHD (ICD-10-AM) have remained consistent from 2005 to 2012, it is possible that the application and interpretation of these codes has changed over time.

## Conclusions

This study demonstrated decreases in acute CHD age-standardised event rates in contrast to increases in chronic CHD age-standardised event rates throughout Victoria from 2005 to 2012. Furthermore, age-standardised event rates for acute and chronic CHD were higher in regional/remote Victoria compared to metropolitan Victoria. These results might reflect a discrepancy in access to appropriate cardiac health-care services between metropolitan and regional/remote Victoria. The changes in acute and chronic CHD age-standardised event rates could be the result of increasing efficacy of primary prevention strategies for CHD without a subsequent increase in the efficacy of secondary prevention strategies. Improvements in primary prevention strategies might serve only to prevent the development of acute types of CHD, namely angina and AMI, thereby shifting CHD burden more towards chronic CHD. It is possible that improved secondary prevention strategies are necessary to attenuate the increasing trend of chronic CHD age-standardised event rates. Furthermore, effective strategies are required to reduce the discrepancy that exists in acute and chronic CHD event rates between regional/remote and metropolitan Victoria.

### Ethics approval and consent to participate

Ethics approval for this study was obtained from La Trobe University Human Ethics Committee (ref: 14/125) and approval to access event admission data from the Victorian Admitted Episodes Dataset was granted by the Victorian Department of Health.

### Consent for publication

Not applicable.

### Availability of data and materials

Data will not be shared. These data have been obtained from the State of Victoria. On receipt of these datasets the authors agreed not to reproduce, distribute or commercialise them, or any product or service derived from incorporating them or part of them (whether or not amounting to copyright reproduction). As recipients, the authors were permitted to publish any analyses of the data, but not the data itself, subject to the confidentiality conditions.

## Abbreviations

AMI: acute myocardial infarction
CHD: coronary heart disease
CI: confidence interval
ICD-10-AM: International Statistical Classification of Disease and Related Health Problems, Tenth Revision, Australian Modification
LGA: local government area
VAED: Victorian Admitted Episodes Dataset
